# General health and working conditions of Flemish primary care professionals

**DOI:** 10.1186/s12875-023-02082-w

**Published:** 2023-06-29

**Authors:** Veerle Buffel, Muhammed Mustafa Sirimsi, Patricia De Vriendt, Dominique Van de Velde, Lies Lahousse

**Affiliations:** 1 Department of Sociology, Centre for Population, Health and Family, University of Antwerp, Antwerp, Belgium; 2 Faculty of Medicine and Health Sciences, Centre for Research and Innovation in Care, University of Antwerp, Antwerp, Belgium; 3 Department of Primary Care and Interdisciplinary Care, Faculty of Medicine and Health Sciences, University of Antwerp, Antwerp, Belgium; 4 Occupational Therapy Department Research Group Health & Care Artevelde University of Applied Sciences, University of Ghent, Ghent, Belgium; 5 Frailty in Ageing (FRIA) Research Group Mental Health and Wellbeing Research Group (MENT) Department Gerontology, Vrije Universiteit Brussel, Brussels, Belgium; 6 Faculty of Medicine and Health Sciences, Department of Rehabilitation Sciences, Research Group Occupational Therapy, Ghent University, Ghent, Belgium; 7 Department of Bioanalysis, Pharmaceutical Care Unit, Faculty of Pharmaceutical Sciences, Ghent University, 9000 Ghent, Belgium

**Keywords:** Primary care sector, Health professionals, General health, Working conditions, Quality of employment

## Abstract

**Background:**

The Quintuple aim explicitly includes ‘health and wellbeing of the care team’ as requirement for the care of patients. Therefore, we examined working conditions, work engagement and health status of professionals active in primary care in Belgium (Flanders), and how these are interrelated.

**Methods:**

Data of the cross-sectional ‘Health professionals survey of the Flemish Primary care academy’ of 2020 were examined. We performed logistic regression analyses to study the relationship between working conditions and self-reported dichotomized health of primary care professionals (sample size = 1033).

**Results:**

The majority of respondents (90%) reported having a good to very good general health and has a strong work engagement. Quality of employment was high, in particular regarding job security and supportive relations with colleagues, while less in terms of proper rewards and job career opportunities. Working as self-employee (vs. as salaried employee), and in a multidisciplinary group practice (vs. other organizational settings) were positively related to health. Work engagement and all dimensions of employment quality were related to general health, but work family balance, proper rewards, and perceived employability were independently positively related to self-reported health.

**Conclusion:**

Nine out of 10 Flemish primary care professionals working in diverse conditions, employment arrangements and organizational settings report good health. Work family balance, proper rewards, and perceived employability were important for primary care professionals’ health, and could provide opportunities to further strengthen the job quality and health of primary care professionals.

**Supplementary Information:**

The online version contains supplementary material available at 10.1186/s12875-023-02082-w.

## Background

Primary care (PC) aims to offer accessible and affordable care to large communities. Strong primary care systems satisfy the curative health needs, health promotion and preventive health needs of the majority of the population and may reduce unnecessary emergency department visits by providing effective referral and discharge systems [1–3].

High quality primary care has to be supported by a good health and social care workforce, collaborating and working under good employment conditions. Striving to a high quality primary care should therefore take into account working conditions, work engagement and well-being and health of the professionals as well. This is also echoed by the transition from the triple aim to the quadruple aim [4] and currently the quintuple aim [5]. The triple aim includes accepted aims of enhancing patient experience, improving population health, and reducing costs for optimization of the health system performance, and is expanded by adding the goal of improving work life of health providers [4] and advancing health equity [5]. Also in the ‘Human experience in healthcare’ approach of the Beryl Institute, the importance of the workforce is highlighted in addition to patients and the community [6]. Workforce well-being and work engagement are considered as powerful ways to improve patient experience [7]. Work engagement is a ‘positive, fulfilling, work-related state of mind [8], which is assumed to be interconnected with job performance, health and well-being [9–11].However current reforms, task redistributions and new or other responsibilities of the PC may change working conditions and put an increasing pressure on PC workers [12, 13]. A lot of flexibility is expected from PC workers, as they have to integrate a continuously increasing amount of information and updated guidelines into their daily work, even more during health crises such as the recent Covid-19 pandemic [13]. Meanwhile, the pandemic also unveiled how resilient primary care is and the intrinsic motivation, self-initiative and commitment among professionals active in the PC [14]. Yet physicians and other members of the health care workforce report widespread burnout and sickness absence, related to multiple occupational factors, personal characteristics and a challenging work environment [15–17].

In recent years, much has been written about well-being, experiences and satisfaction of patients in primary care [18] but little attention has been paid on well-being, health, and working conditions of health professionals [19]. There is increasing recognition that patient safety, satisfaction and access to high quality healthcare is linked to the well-being of health professionals [20–22] and it is known that well-being is strongly related to health, and in particular to self-rated health status [23, 24]. In addition, previous research has confirmed a strong relation between working conditions and health outcomes among the general working population [25–29]. However, until now, most studies among health professionals are focusing either on working conditions or on health and safety of health professionals, but not on the relation between both. In addition, they are focusing on health professionals in general [30], specific professions (e.g. general practitioners (GPs) [31, 32], nurses [22, 33], etc.) or professionals working in the institutionalized health sector (e.g. within hospitals [34]), but not on the primary care sector in its entirety, across the multiple disciplines involved.

The PC sector has a diverse landscape of health and social care professionals, including general practitioners, nurses, physiotherapists, dieticians, pharmacists, social care workers, midwives, podiatrists, first-line psychologists, occupational therapists, family care assistants, speech therapists, dentists and supporting staff. PC professionals are also working in different organizational settings (working solo or in group, mono- or multidisciplinary, etc.) and vary in employment arrangement (salaried employment vs. self-employment; or a mix of both), which may be related to a variation in quality of working conditions, work engagement and health. Knowing which aspects of quality of employment are important for PC professionals’ health and detecting where improvements can be made, is of the utmost importance for the working life and health of PC professionals, and thus for the quality of PC in itself.

The aim of this study is therefore threefold: first, we perform a mapping of health professionals active in Flanders, the Dutch-speaking PC sector in Belgium, including their sociodemographic and job characteristics; second, we investigate current health and working conditions of PC professionals, and third, we study whether their working conditions and work engagement are related to their general health status.

## Methodology

### Data

The ‘health professionals survey of the Flemish Primary Care Academy’ (PCA) (a network for research and education aimed at primary care in Flanders and Brussels, consisting of 4 universities and 6 colleges) collected information through a cross-sectional online survey about sociodemographics, working conditions and general health of health and social care professionals active in the Flemish PC sector (see Supplementary file S1 for the English translation of the short online survey). After a piloting phase in May 2020, the data were collected between June and September 2020, through standardized online questionnaires in Dutch using Lime software. At the beginning of the survey, participants were informed and asked to complete the survey on their conditions before the COVID-19 pandemic.

Respondents were recruited in two ways. First in a general way, through advertising on social media (Linked in®, Twitter®, Facebook®), the website and contact list of the PCA and second, by a more targeted approach by actively contacting professional associations and other supportive organizations of PC professionals and asking them to spread the survey by mailing their members and placing announcements on their website and newsletters. The recruitment process was monitored and additional efforts taken to reach underrepresented professions active in the PC sector.

Study participants had to meet the following criteria: (i) being employed in one (or more) of the 60 PC zones (employed in Flanders), (ii) being 18 years or above, (iii) accepting to answer the study questionnaire, and (iv) being able to read and understand Dutch. Ethical approval was obtained through the ethical committee of the University Hospital of Antwerp (registration code: B300201942302 and reference number: 19/42/461).

#### Measures

##### Health conditions

The principal outcome is *self-rated health* (SRH) which reflects how respondents rate their health, answering a single item on a 5-point scale ranging from "very good" [1] to "very poor" [5]. It was shown that such self-ratings represent a source of reliable and valid data on health status [35, 36]. For interpretability and comparability with other studies, the variable was dichotomized. Categories fair, poor and very poor were classified into poor self-rated health and good to very good in good self-rated health [37]. Additional health variables are *‘limited in daily activities due to disability or illness’* (0 = no; 1 = (strongly) limited) and *‘long sickness absence’* (if people are 20 or more days a year absent because of health problems) [38].

##### Sociodemographics

As sociodemographic variables, we include age (categorized per 15 years), biological sex, migration background, household composition, educational level, and working place (by province).

##### Job characteristics, working conditions and work engagement

All fifteen *professions* recognized as PC professionals by the Flemish government were addressed: general practitioner, nurse, physiotherapist, dietician, podiatrist, midwife, first line psychologist, dentist, social care worker, occupational therapist, pharmacist, family care assistants (also called ‘domestic aid’), speech therapist, and care supporting staff. The survey also provided a category ‘other’ including for example e-health coaches, diabetes educators, coordinators of community health centers or case managers.

The *organizational settings* in which the PC professionals are working, were questioned: in a solo-practice, a monodisciplinary group practice, multidisciplinary group practice, at patients’ home, in social services (e.g. Centre for General wellbeing, Public Centre for Social Welfare, mutuality) or other (e.g. Centre for Mental wellbeing).

The working conditions questioned in the survey were derived from the European working conditions survey. Questions were based on the theoretical framework outlined by Eurofound [38] to measure job quality. This framework was developed as an accurate measure of contemporary aspects of job quality and of which the empirical use is demonstrated in studies on the link between working conditions and health as well [27, 39]. Quality of employment can be seen as a multidimensional concept reflecting the concepts of contract security, employability, employment relations, income and rights, and working time. These concepts are subsequently measured in our study by *contract type*, with a relevant distinction for PC health professionals between [1] employed with a contract of unlimited duration, [2] employed with a contract of a limited duration, [3] self-employed or [4] a mix between self-employed and employed. In the Flemish primary care sector these employment arrangements broadly overlap with the two financing systems in which primary care professionals works [40]: those who are employed are mostly embedded in a capitation system, while the self-employed in a fee-for-service (FFS) system. However, this does not have to be the case, and depends on the type of profession. For example, a home nurse can be employed or self-employed, but will always work in a FFS system [41], while a nurse in a primary care practice with a capitation system will predominantly work as an employee [42]. GPs working in a FFS system, will always be self-employed, while GPs working in a capitation system will mostly work as a salaried worker, but can also be self-employed [42, 43]. Also *perceived job security* is included, by asking the respondents how likely it is that they may lose their job in the next 6 months (ranging from 1 ‘very likely’ to 5 ‘very unlikely’). *Career opportunities and perceived employability (or also called labor market security)* are considered as indicators of employability and *good relations with colleagues* and *job recognition* as proxies for supportive employment relations. These variables are respectively measured by the degree to which the respondents agree with the following statements (Likert scale: 1 = strongly disagree to 5 = strongly agree): My job offers good prospects for career advancement; It would be easy for me to find a job of similar salary in the event of losing or leaving my current job; I generally get on well with my work colleagues; and I receive the recognition I deserve for my work.

For the variable *proper rewards,* respondents were asked whether they agree with the following statement: Considering all my efforts and achievements in my job, I feel I get paid appropriately (Likert scale). Proper rewards are used as proxy for income and rights, as no objective measures of income and rights were included.

*Working time* is based on the number of hours worked per week and consists of the following categories: “part-time” (< 35 h per week), “full-time”, and “long hours” (> 40 h per week). Another relevant indicator related to working time is the degree (from 1 = very bad to 5 = very good) to which working hours fit in with family or social commitments outside work (defined as *work-life balance*) as health care professionals have often varying working hours to ensure continuity of care, which may complicate a good balance between work and family responsibilities [22].

*Work engagement* is measured by the Utrecht Work Engagement Scale (UWES-3 scale) which is also validated in Belgium (Flanders) [44]. The scale consists of three items corresponding to those three dimensions of work engagement [8]: (1) “At my work, I feel bursting with energy” (vigor); (2) “I am enthusiastic about my job” (dedication); (3) “I am immersed in my work” (absorption). The scale ranges from 1 to 5 with a higher score referring to a higher level of work engagement. The Cronbach’s’ alpha is 0.8.

##### Analysis

First, a description of included PC professionals is given in terms of sociodemographic characteristics. Thereafter, the health status, working conditions, job quality and work engagement are described. Chi-square or Fisher’s Exact tests were used to determine differences in working conditions between the various professions. Second, the relationships of type of profession, working conditions, job quality, and work engagement with dichotomized subjective health status of PC professionals was evaluated by logistic regression analyses. The strength of the associations were presented by unadjusted (or crude) odds ratio’s (OR) and adjusted OR (adjusting for the variables which were significantly related to subjective health in the univariable analyses (see unadjusted ORs)). Statistical significance is set at p ≤ 0.05. Finally, differences in job quality and work engagement across the various professions and working conditions were explored (using Anova and post hoc Bonferroni tests).

## Results

**Fig. 1:**
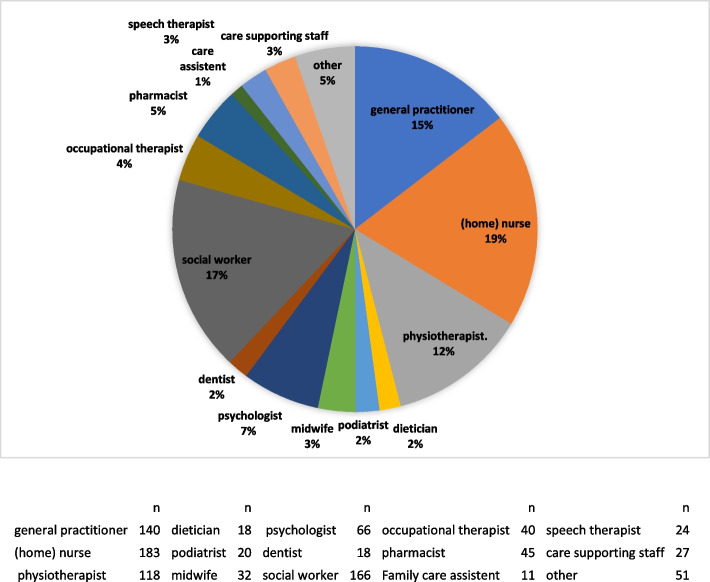
Distribution (%) of the different types of primary care professionals in the ‘Primary health

In total, 1033 PC professionals signed the informed consent of the survey and 80% of these respondents (*N* = 826) provided information on all variables included in this study. The majority was female (77.6%) and born in Belgium (95.2%). One third of PC professionals were between 35 and 50 years old (35.7%), almost half were cohabiting (or married) with partner and children (48.8%), and almost half achieved a university degree (46.5%) (Table 1). All fifteen professions recognized as PC professionals by the Flemish government were represented (see Fig. 1). The largest participating profession group were nurses (19.1%), followed by social workers (17.3%), GPs (14.6%) and physiotherapists (12.3%). Respondents geographically represented all five provinces in Flanders and Brussels, with the highest percentage of PC professionals in Antwerp (30.9%), followed by East- (26.1%) and West-Flanders (20.9%).

**Table 1:** Description of the sample of primary care health professionals in Flanders: Sociodemographic profile, health conditions, organizational setting and employment conditions

	N tot	% missing	n	%
SOCIODEMOGRAPHIC PROFILE				
**Sex**	1033	0.00		
Men			231	22.36
Women			802	77.64
**Age (in years)**	1030	0.29		
21–35			350	33.98
36–50			368	35.73
51–75			312	30.29
**Migration background**	1030	0.29		
Born in Belgium			981	95.24
Born outside Belgium			49	4.76
**Household structure**	1026			
Couple without children at home			310	30.30
Couple with children at home			499	48.78
Living together but not with partner			85	8.31
Living alone			129	12.61
**Geographical area (province)**	939	9.10		
Brussels-Capital Region			21	2.24
Flemish-Brabant			108	11.50
Antwerp			290	30.88
Limburg			79	8.41
West-Flanders			196	20.87
East-Flanders			245	26.09
**Highest educational level**	1032	0.10		
Secondary school or less			39	3.78
Non-university higher education (short type)			410	39.69
Non-university higher education (long type)/short university study (only bachelor)			103	9.97
University studies			480	46.47
HEALTH CONDITIONS	932	9.78		
(very) good general health			826	88.60
(strong) limitations in daily activities due to disability or illness			147	15.80
Long sickness absense (> = 20 days a year)			131	14.10
ORGANISATIONAL SETTING				
**Organisational setting**	938	9.20		
Professional setting: solo			162	17.27
Group-monodisciplinary			196	20.90
Group-multidisciplinary			214	22.81
Care at home of patients			163	17.38
Social service center			144	15.35
Other			59	6.29
EMPLOYMENT CONDITIONS				
**Type of contract (employment arrangement)**	939	9.10		
Employed with unlimited contract			441	46.96
Employed with limited contract			30	3.19
Self-employed			339	36.10
Mix			129	13.74
**Working hours**				
Parttime (< 35 h a week)	940	9.00	351	37.34
Fulltime (35 to 40 h a week)			327	34.79
More than fulltime (> 40 h a week)			262	27.87
**Job quality scales (1–5)**			**Mean**	**SD**
Work life balance	901	12.78	3.59	1.01
Perceived job security	876	15.20	4.19	0.89
Proper rewards	876	15.20	3.02	1.18
Career opportunities	876	15.20	3.09	1.02
Perceived employability	875	15.30	3.12	1.13
Supportive relations with colleagues	876	15.20	4.29	0.69
Job recognition	876	15.20	3.44	0.99
**Work engagement** (1–5)	875	15.30	4.10	0.52
Total N of the analytical sample^a^	826	20.00		

*SD* Standard deviation; ^a^The analytical sample will be used for the logistic regression analyses

Nine out of ten (88.6%) of PC professionals reported to have a good to very good general health. A small proportion, 15.8% answered to be (strongly) limited in daily activities due to disability or illness. Similarly, 14.1% was absent for 20 days or more during the last year because of sickness, while more than half of the professionals (50.5%) was not absent for a single day during the last year. The average number of sick days was 14 days a year, with large differences between professionals (lowest among dentists, GPs, podiatrist). The sickness absenteeism also differed by organizational setting or financing systems with substantially lower (*p* < 0.001) absenteeism among professionals working solo and self-employed than professionals working in social services or as salaried employee, respectively.

More than 40% is working in a group practice, of which just over half in a multidisciplinary team (Table 1). Especially younger health professionals were active in group practices (51.6% vs. 40.5% and 39.6%, not presented in the table) and male professionals in solo-practices (28.2% of all male respondents, while only 15.3% of female respondents). In particular care supporting staff, dieticians, podiatrists, psychologists and GPs are active in a multidisciplinary team, while dentists, pharmacists, physiotherapists, midwives and speech therapists are mostly working solo or in a monodisciplinary team (see Supplementary files S2 and S3).

One third of professionals is working part-time (37.3%), another third fulltime (between 35 and 40 h a week: 34.8%), and almost one third (27.8%) of respondents -especially GPs (65.0%), physiotherapist (55.2%) and pharmacists (48.8%)- is working more than 40 h a week. Working hours varied strongly by organizational setting and employment arrangement (see S2), with higher percentages of PC professionals working more than 40 h a week among those working in a monodisciplinary team (54.3%) or solo-workers (44.0%) and among self-employed professionals (57.7%).

Almost half of PC professionals is working as a salaried employee (47.2%), of which only a minority is employed with a contract of limited duration (3.2%). Moreover, a large part of PC professionals is self-employed, especially GPs (70.4%, S2), physiotherapists (72.9%), and dentists (100%).

The highest average score on the quality of employment scales can be found on supportive relations with colleagues (x̄[sd] = 4.19[0.89]) and perceived job security (4.29[0.69]), while the lowest on proper rewards (3.02[1.18]) and career opportunities (3.09[1.02]). The average score on the work engagement scale is quite high (4.10[0.54]).

The results of the logistic regression analyses presented in Table 2- show that a good self-perceived general health is significantly more likely among those born in Belgium (vs. with a migration background: 0.454[0.096]) and among those with university studies compared to lowest two educational levels (0.234[0,005] and 0.442[0.001]). Compared to GPs, nurses (0.449[0.052]), podiatrists (0.324[0.087]), social workers (0.402[0.027]), and family care assistants (0.108[0.008]) have a lower likelihood to report a good general health.

**Table 2:** Logistic regression analyses: Sociodemographic and job characteristics and working conditions related to ‘having a good general health’ (0/1) among primary health care professionals

	OR	p		AOR	p	
**Sex** (ref. men)						
Women	0.882	0.650		0.898	0.748	
**Age** (ref. 21–35 years old)		0.147			0.261	
36–50 years old	0.606	0.067	a	0.614	0.112	
51–75 years old	0.626	0.103		0.637	0.207	
**Migration background** (ref. born in Belgium)						
Born outside Belgium	0.454	0.096	a	0.258	0.014	*
**Highest educational level** (ref. university studies)		0.001	***		0.129	*
Secondary school or less	0.234	0.005	**	0.481	0.338	
Non-university higher education (short type)	0.442	0.001	***	0.500	0.105	
Non-university higher education (long type)	1.239	0.668		1.328	0.635	
Or short university study (only bachelor)						
**Household structure** (ref. couple with children at home)		0.496			0.067	a
Couple without children at home	1.361	0.245		1.489	0.179	
Living together but not with partner	0.782	0.512		0.471	0.081	a
Living alone	1.216	0.594		1.547	0.292	
**Profession** (ref. GP)		0.009	**		0.403	
(home) Nurse	0.449	0.052	a	0.943	0.928	
Physiotherapist	2.973	0.109		5.733	0.022	*
Dietician						
Podiatrist	0.324	0.087	a	0.617	0.546	
Midwife						
Psychologist	0.517	0.200		0.617	0.427	
Dentist	0.378	0.180		0.385	0.251	
Social worker	0.402	0.027	*	0.922	0.914	
Occupational therapist	0.432	0.137		0.830	0.825	
Pharmacist	3.000	0.305		4.028	0.221	
Family care assistent	0.108	0.008	**	0.590	0.667	
Speech therapist	0.459	0.277		1.361	0.720	
Care supporting staff	1.622	0.655		1.801	0.620	
Other	1.103	0.866		1.228	0.796	
**Organizational setting** (ref. group-multidisciplinary)		0.010	**		0.825	
Group-monodisciplinary	1.355	0.455		1.594	0.329	
Solo	0.817	0.592		1.099	0.846	
At home of the patients	0.505	0.053	a	0.794	0.593	
Social service center	0.449	0.022	*	0.922	0.871	
Other	0.366	0.027	*	0.874	0.798	
**Type of contract/ employment arrangement** (ref. self-employed)		0.048	*		0.486	
Employed with unlimited contract	0.660	0.082	a	0.803	0.612	
Mix (employed and self-employed)	1.504	0.322		1.483	0.421	
**Working hours** (ref. fulltime)		0.215			0.209	
Parttime	0.930	0.772		0.902	0.725	
More than fulltime (> = 40 h. a week)	1.518	0.162		1.902	0.125	
**Work life balance**	1.390	0.001	***	1.452	0.006	**
**Perceived job security**	1.340	0.010	**	1.248	0.105	
**Proper rewards**	1.353	0.002	**	1.331	0.030	*
**Career opportunities**	1.232	0.050	*	0.953	0.735	
**Perceived employability**	1.388	0.001	***	1.345	0.012	*
**Supportive relations with colleagues**	1.631	0.001	***	1.283	0.180	
**Job recognition**	1.485	0.001	***	1.198	0.209	
**Work engagement**	1.929	0.001	***	1.437	0.117	

*OR* oddsratio; *AOR* = adjusted oddsratio: adjusted for educational level. type of profession. organizational setting. type of contract. all employment quality scales and work engagement; p = *p*-value: ^a^ *p* < 0.1 (marginally significant); * *p* < 0.05; ** *p* < 0.01; *** 0.001; *N* = 826

Working in a multidisciplinary group (vs. at home of patients (0.505[0.053]), social services (0.449[0.022]) or somewhere else (0.366[0.027]) and as self-employee) (vs. as a salaried employee (0.660[0.082])) are positively related to the health status of PC professionals. Working hours had no significant effect on the general health status of professionals., but a good fit between their working hours and responsibilities outside their job (good work life balance) was significantly and positively (1.390[0.001]) related to good health status. Also the other quality of employment scales (job security, proper rewards, career opportunities, perceived employability, supportive relations with colleagues and job recognition) and work engagement were positively related to the health status of PC professionals. Job recognition (*r* = 0.303, *p* < 0.001) was most strongly correlated to work engagement, followed by work-life balance (*r* = 0.174, *p* < 0.001) and career opportunities (*r* = 0.170, 0.100), while proper rewards and perceived employability were not significantly related to work engagement (see Supplementary file S3).

In a next step, we adjusted the ORs for the variables significantly (at *p* < 0.05) related to health in the previous step (educational level, type of profession, organizational setting, employment arrangement, the quality of employment scales and work engagement). With regard to the quality of employment, work-life balance (1.452[0.006]), proper rewards (1.331[0.030]), and perceived employability (1.345[0.012]), were independently positive related to self-reported general health.

Regarding job quality dimensions (Table 3), work life balance and proper rewards were especially low rated among professionals who work 40 h a week or more (mean[sd] = 3.02[1.06] and 2.65[1.19]), are self-employed (3.29[1.09], 2.70[1.18]) and/or working solo (3.38[1.09]; 2.75[1.17]) or in a mono disciplinary practice (3.31[1.02]; 2.78[1.21]); and these employment conditions were also strongly clustered (see S3). In contrast, work engagement scored highest among professionals who work 40 h a week or more (4.18[0.51], are self-employed (4.19[0.50]), and working in a mono-disciplinary practice (4.20[0.50]).

**Table 3:** Employment quality by working conditions (Employment arrangement, organizational setting and working hours) and type of profession

	**work** **engagement**		**work life** **balance**		**Proper** **rewards**		**perceived** **employability**		**supportive relations** **with colleagues**		**Job security**		**carreer** **opportunities**		**job** **recognition**	
	mean	sd	mean	sd	mean	sd	mean	sd	mean	sd	mean	sd	mean	sd	mean	sd
**Employment arrangement**		***		***		***				***		***				
Self-employed	4.19	0.50	3.29	1.08	2.70	1.18	3.10	1.19	4.15	0.77	4.41	0.72	3.13	1.09	3.39	1.03
Employed	4.03	0.53	3.84	0.91	3.27	1.10	3.12	1.07	4.38	0.62	4.08	0.90	3.05	0.95	3.52	0.93
Mix	4.09	0.53	3.52	0.98	2.88	1.16	3.24	1.19	4.33	0.64	4.03	1.06	3.02	1.01	3.36	1.05
**Organisational setting**		**		***		***		***		***		**		***		***
Group-multidiscipl	4.08	0.52	3.64	1.00	3.25	1.19	3.42	1.10	4.44	0.65	4.19	0.91	3.18	1.05	3.66	1.01
Group-monodiscipl	4.20	0.50	3.31	1.02	2.78	1.21	3.30	1.13	4.39	0.62	4.36	0.78	3.18	1.08	3.41	1.03
Solo	4.12	0.51	3.38	1.09	2.75	1.17	2.82	1.26	3.92	0.76	4.27	0.87	2.83	1.02	3.27	0.98
Care at home of patient	4.15	0.54	3.66	1.02	2.96	1.11	3.10	1.03	4.29	0.70	4.16	0.86	3.25	0.91	3.49	0.90
Social service	4.00	0.50	4.06	0.79	3.32	1.04	2.79	0.93	4.37	0.66	4.04	0.88	2.98	0.91	3.50	0.88
Other	3.93	0.62	3.49	0.92	2.78	1.15	3.27	1.16	4.18	0.53	3.96	1.09	2.78	1.04	2.98	1.08
**Working hours** (a week)		**		***		***				**		**				
Parttime < 35 h	4.05	0.57	3.88	0.87	3.12	1.13	3.11	1.14	4.23	0.69	4.08	0.92	3.00	0.92	3.47	0.96
Fulltime 35–40 h	4.09	0.48	3.73	0.95	3.16	1.15	3.16	1.07	4.38	0.62	4.21	0.87	3.16	1.02	3.50	0.97
Fulltime 40 + h	4.18	0.51	3.02	1.06	2.65	1.19	3.11	1.20	4.24	0.75	4.35	0.80	3.08	1.13	3.36	1.04
**Profession**		***		***		***		***		**		***		***		*
GP	4.09	0.48	3.17	1.04	3.46	1.16	3.46	1.34	4.40	0.70	4.58	0.68	3.43	1.09	3.69	1.02
(home) nurse	4.22	0.51	3.57	1.00	2.84	1.18	3.40	1.07	4.37	0.60	4.32	0.78	3.26	0.96	3.45	0.94
Physiotherapist	4.19	0.49	3.30	1.01	2.27	1.05	3.07	0.97	4.21	0.73	4.16	0.83	2.90	1.06	3.29	1.02
Podiatrist	3.87	0.63	3.35	0.88	3.05	1.05	2.70	0.92	3.95	0.51	4.30	0.73	3.05	0.89	3.40	0.88
Psychologist	4.04	0.46	3.88	0.89	2.71	1.15	2.92	1.15	4.14	0.68	3.86	1.09	2.93	0.94	3.39	1.03
Dentist	3.98	0.36	3.59	1.00	3.65	1.06	3.35	1.58	4.12	0.86	4.35	1.00	3.53	0.87	3.65	1.00
Social worker	3.97	0.49	4.08	0.78	3.40	1.06	2.81	0.93	4.31	0.69	4.08	0.87	2.94	0.95	3.50	0.91
Occupational therapist	4.06	0.61	4.13	0.70	3.18	0.90	2.74	1.13	4.39	0.50	3.79	1.07	2.97	1.03	3.34	0.91
Pharmacist	3.96	0.56	3.05	1.01	3.13	1.02	3.74	1.06	4.37	0.75	4.24	0.75	2.63	0.97	3.03	1.03
Family care assistent	3.90	0.57	3.29	1.25	2.57	1.13	3.29	0.95	3.86	0.69	3.71	0.76	2.43	0.79	2.86	1.35
Speech therapist	4.15	0.76	3.05	1.32	2.55	1.15	2.80	1.36	4.25	0.72	4.45	0.69	2.65	0.93	3.35	1.09
Care supporting staff	4.00	0.65	4.14	0.85	3.67	1.02	3.24	1.04	4.52	0.51	4.19	0.68	3.19	1.08	3.76	1.00
Other	4.26	0.51	3.58	1.01	2.77	1.12	2.88	1.03	4.10	0.82	4.01	1.06	3.07	0.98	3.44	0.99

Anova test; * *p* < 0.01 ** *p* < 0.05 *** *p* < 0.001; *N* = 826

Professionals working in multidisciplinary group practice scored highest on perceived employability (3.42[1.10]) and supportive relations with colleagues (4.44[0.65]). With the exception of supportive relations with colleagues, the employment quality variables also showed significant variation across the different types of professions. On average, work life balance is rated relatively low among GPs (3.17[1.04]), pharmacists (3.05[1.01] and speech therapists (3.05[1.32]). Speech therapists also scored low on perceived employability (2.80[1.36]) and proper rewards (2.55[1.15]), while GPs (3.46[1.34]) and pharmacists (3.74[1.06]) are scoring relatively high on perceived employability, and GPs (3.46[1.16]) -together with dentists (3.65[1.06]), care supporting staff (3.67[1.02]) and social workers (3.40[1.06])- also high on proper rewards. In contrast, physiotherapists (2.27[1.05]), family care assistants (2.57[1.13]) and first line psychologists (2.71[1.15]) are scoring low on proper rewards, and podiatrists (2.70[0.92]) and occupational therapists (2.74[1.13]) low on perceived employability. Work engagement was highest among physiotherapists (4.22[0.51]) and (home) nurses (4.19[0.49]), while lowest among podiatrists (3.87[0.63]), social workers (3.97[0.49]), pharmacists (3.96[0.56]), and family care assistants (3.90[0.57]).

## Discussion and conclusion

In this study, we examined the health status, working conditions and work engagement of PC professionals in Flanders (Belgium). Approximately 90% of the PC professionals reported their health as good to very good, which is comparable to the proportion among the general working population of the same age range in Flanders in 2018 [45], but almost 20% higher than the overall Belgian or European working population surveyed by Eurofound during the same period (spring–summer of 2020) [46]. An average of 14 sick days a year was in line with 13 sick days among Belgian employees in the private sector (based on the information of 750 000 employees) [47]. More than half of our included PC professionals was never absent due to sickness over the past year. If we stratify on the salaried employees, the presenteeism among PC professionals (34%) is similar to that among federal officials (33%) [48] or employees in the social sector (35%), but lower than among those working in the private sector (61%) in 2019 [49].

PC professionals are working in very different organizational settings and employment arrangement [50]. In line with several other European countries (Bulgaria, the Czech Republic, Denmark, Estonia, Germany, Hungary, Ireland, Italy, Latvia, Luxembourg, the Netherlands, Norway, Romania, Slovakia, Switzerland, Turkey and the UK), the dominant employment status of PC professionals, in particular GPs, is self-employment [50]. Thirty six percent of PC professionals was self-employed or employed in a mixed system in comparison to 9.3% and 8.6% among the general Flemish and Belgian working population in 2020 [51], while a much smaller part is working with a temporary contract (3% vs. 8% in 2020 in Belgium) [52]. On average, the percentage of PC professionals working 40 h or more a week (28%) is very similar to that of the general Belgian (27%) and European working population (29%) (based on round 2 of Eurofound e-survey, 2020), but the percentage (37%) of part-time working PC professionals is almost 10% higher [46]. However, this can be mainly ascribed to the fact that the majority of PC professionals is female (77%), and among female PC professionals the percentage of working parttime is the same as among the general working population (around 42%). In terms of employment quality, the PC professionals are scoring relatively high, especially on ‘job security’ (4.19[0.89]) and ‘supportive relations with colleagues’(4.29[0.69]). While the general working population in Belgium (4.29[1.05]) and Europe (4.15[1.09]) also score very high on job security (based on round 2 of Eurofound e-survey, 2020), this was less the case for support from colleagues (average score in Belgium: 3.51[0.99]; in Europe: 3.54[1.07]) [33].

Some differences in employment conditions and quality may explain differences in perceived general health among PC professionals, with GPs reporting a significantly better general health than family care assistants, and also people working in a multidisciplinary team compared to those working in other organizational settings. Self-employment is also positively related to a good health (compared to employment). The independence that comes with self-employment may give autonomy and flexibility, which may lead to a better self-perceived health [53]. However, it is also possible that professionals with a poorer health prefer working as a salaried employee, which provides a better social protection (referring to the health selection effect [53]). Some PC professions also do not have the option to choose between employment arrangement (self-employed or salaried employee). For example, all dentists in our sample are self-employed, while family care assistants, care supporting staff and social workers are commonly employed. If dentists aim to work as a salaried employee in a primary care practice with a capitation system, this is very difficult, as the capitation system for primary care in Belgium does not cover dental care [42, 54].

Working parttime, fulltime or more than fulltime, was not related to self-perceived health status, although professionals working 40 h or more a week reported worse work life balance, while the latter was positively related to a good general health. This might reflect reverse causality, as their good health may allow them to work many hours. This may compensate for the potential negative effect of a poor work family balance. Also all other dimensions of employment quality (career opportunities, job security, job recognition, etc.), were positively related to general health, but only work life balance, proper rewards, and perceived employability, were significantly related to PC professionals’ general health, taking the other factors and work engagement into account. The importance of these dimensions are described earlier as facilitating factors for professional fulfilment as described in the Stanford model of professional fulfilment [55]. In addition, these dimensions of job quality were also found in previous European research among nurses [56, 57] and GPs [34, 58], and not only in terms of health, but also for job satisfaction and as motivation for leaving or staying in the job [58, 59].

Finally, a lot of variation was observed among these important aspects of job quality and work engagement across professions and working conditions. This makes it challenging to assess which working conditions are ideal in terms of job quality, which are in turn important for PC professionals’ health. However, we were able to indicate among which working conditions and professions these job quality aspects were rated as good to very good, and also where improvements could be made. Work life balance and proper rewards were especially worse among PC professionals who are self-employed, working more than 40 h a week, and working solo or in a monodisciplinary group practice, indicating that investing in a supportive context, where PC professionals across the different disciplines can work together, with special attention for work-life balance, is important. A concrete example may be the provision of (more) financial compensation for administrative and practice support [59, 60]. Employability and supportive relations with colleagues were perceived as very good especially in multidisciplinary group practices. Although multidisciplinary group practices are often encouraged for the improvement of integrated healthcare of patients [50], it may thus also be positive for the working life and wellbeing of PC professionals themselves.

### Strengths and limitations

This is the first Flemish study and one of the first in Europe that has investigated the working conditions, job quality and general health of professionals active in PC. The originality of our study lies in the inclusion of all PC professions. However, also some limitations of this work should be addressed. First, it is a cross-sectional survey. As a result, we are unable to disentangle whether the working conditions significantly associated with good general health, causally lead to improved subjective health. People with a better health may perceive their working conditions as better (reporting bias), may be more able to do (more) work (reverse causality), or may obtain or retain jobs with better job conditions including higher wages.

Second, the survey made use of a convenience sample as there is no clear sample frame for the PC sector available. However, there was a strong monitory during the sampling process and strategic recruitment to cover all professional and guarantee geographical dispersion. The sociodemographic profile of the PC professionals in our sample (the majority was female, born in Belgian, between 36–50 years old, cohabitating with partner and children and highly educated) was also comparable with that of general health care providers working and living in Belgium at the beginning of 2020 [45]. There was some overrepresentation of GPs, podiatrists and psychologists in our survey, and an underrepresentation of dentists, pharmacists, midwives, and speech or occupational therapists. As a result, for some professions, the sample may be less representative, and therefore no strong conclusions could be made for all professions present in primary care. Future research however could further highlight differences between professions and provide more insight in how different professions deal with the same issues.

In addition, selection bias cannot be excluded, as it may be likely that health care providers who experienced better working conditions and a better health were more likely to respond to our invitation to participate in the study, and professionals with a more disadvantaged socioeconomic background, limited access to the internet or with a very high work load or poor health were generally less likely to participate in surveys.

There was also a relatively large drop out during the survey. The survey was held in the beginning of the COVID-19 pandemic, which could cause health care professionals with a higher workload not to participate or fully complete the survey and those with a temporary lower workload to be too optimistic about their working conditions [13]. Nevertheless, the survey (May-sept 2020) period was between two waves of the COVID-19 pandemic in Flanders, and the drop out was comparable to other survey research in the Flemish health care sector in non-COVID times [61]. Moreover, we have limited the length of the survey by limiting additional questions about intrinsic job characteristics. Although more information about psychological demands, level of autonomy, flexibility, representation and skill discretion could have been interesting since these relate to health outcomes, also among health professionals [62]. High job demands in combination with a low level of control and imbalances between efforts and rewards are moreover important predictors of burn-outs among health professionals [56, 63]. Therefore, further research could further investigate extrinsic and intrinsic job characteristics and how their combination impacts health outcomes of PC professionals over time.

## Conclusion

To conclude, work life balance, proper rewards, and perceived employability re all significantly associated with the general health of PC professionals. It is consequently important to recognize the importance of these job resources for the general health status of PC professionals, in particular in the current context of increasing responsibilities, organizational changes and shortness of professionals within the PC sector. With our study we were also able to detect where further improvements could be made to potentially strengthen the job quality and health of PC professionals in Flanders.

## Supplementary Information

**Additional file 1.** 

**Additional file 2.** 

**Additional file 3.** 

**Additional file 4.** 

## Data Availability

The datasets used and/or analysed during the current study are available from the corresponding author on reasonable request.

## Abbreviations

PC: Primary care
GP: General practitioner
FFS: Fee-for-service
PCA: Primary Care Academy
SRH: Self-rated health
OR: Odds ratio
