# Supplementary material for: General health and working conditions of Flemish primary care professionals
Source: BMC Prim Care. 2023 Jun 29;24:133. doi: 10.1186/s12875-023-02082-w (PMC10308612; doi:10.1186/s12875-023-02082-w)
Supplement: Supplementary file 1 — Additional file 1. [file 12875_2023_2082_MOESM1_ESM.docx]

**SUPPLEMENTARY FILE**

**S1.** English translation of the short online survey of the Primary Care Academy

1. **Are you:**

- A man
- A woman

1. **In what year were you born?**

**_ _ _ _**

1. **In what country were you born?**

- In Belgium
- In another European country than Belgium
- In a non-European country

1. **What is your current family structure?**

- Cohabiting with partner and without resident children
- Cohabitating with partner and resident children
- Cohabiting, but not a with partner
- Living alone but with a partner
- Living alone and being single
- Other

1. **What is the highest level of training or education you have successfully completed?**

- Elementary school not completed
- Primary school diploma
- Lower secondary education: lower vocational secondary education (BSO)
- Lower secondary education: lower technical secondary education (TSO)
- Lower secondary education: lower general secondary education (ASO)
- Upper secondary education: diploma in vocational secondary education (BSO)
- Upper secondary education: diploma in technical secondary education (TSO)
- Higher secondary education: diploma of general secondary education (ASO)
- Higher non-university education of the short type (2-3 years)
- Higher non-university education of the long type (4 years)
- University education: candidate or bachelor
- University education: licentiate or master's degree
- University education: doctorate

***In the following questions, we are interested in your main activity as primary care providers/actors.***

1. **As what health care provider or professional are you employed in primary care?**

- general practitioner
- (home) nurse/nurse practitioner
- physical therapist/kinesist
- dietician
- podiatrist
- midwife
- primary care psychologist
- dentist
- social worker
- occupational therapist
- pharmacist
- audiologist/audiologist,
- healthcare professional
- speech therapist
- other

1. **In what professional setting(s) are you primarily employed?**

- solo (practice)
- group practice (monodisciplinary)
- group practice (multidisciplinary)
- home health care provider
- in a center for mental health
- other

1. **Under which funding system do you primarily work?**

- salaried worker (contract of indefinite duration)
- salaried worker (contract of limited duration)
- employed via temporary employment agency or secondment agency
- self-employed (per consultation)
- in training/internship
- other

1. **Under what funding system do you work in the second instance?**

- salaried worker (contract of indefinite duration)
- salaried worker (contract of limited duration)
- employed via temporary employment agency or secondment agency
- self-employed (per consultation)
- in training/internship
- other

1. **On average, how many hours do you work per week as a primary care provider?**

**_ _**

1. **Since when have you been active in primary care?**

**_ _ _ _**

1. **In which municipality are you most active as a primary care provider? Enter your zip code here.**

**_ _ _ _**

1. **How is your health in general?** Would you say that this is ... (fill in)

- Very good
- Good
- Fair
- Poor
- Very poor

1. **Are you limited in your daily activities due to an illness or health problem?**

- Yes, severely limited
- Yes, somewhat limited
- No, not limited at all

1. **During the past 12 months, how many total days were you absent from work due to sick leave or health-related leave?**

**_ _** number of working days in the last 12 months (give a best possible estimate)

1. **Which of the following statements best describes your skills in your own work??**

- I need further training to do my tasks well
- My current skills match well with my tasks
- I have the skills to handle a more demanding set of tasks

1. **In general, to what extent can you combine your working hours with your family and social commitments outside work?**

- Very well
- Good
- Not very well
- Not at all

1. **Overall, are you very satisfied, satisfied, not very satisfied or not at all satisfied with the working conditions of your main paid job?**

- Very satisfied
- Satisfied
- Not very satisfied
- Not at all satisfied

1. **To what extent do you agree or disagree with the following statements about your current job?**

19.a. Considering all my efforts and achievements in my job, I feel I get paid appropriately

19.b. My job offers good prospects for career advancement

19.c. I receive the recognition I deserve for my work

19.d. I generally get on well with my work colleagues

19.e I might lose my job in the next 6 months

19.f If I were to lose or quit my current job, it would be easy for me to find a job of similar salary.

- Strongly agree
- Tend to agree
- Neither agree nor disagree
- Tend to disagree
- Strongly disagree

1. **The following statements are about how you feel about your job. For each statement, please tell me how often you feel this way…**

20.a. At my work I feel full of energy

20.b. I am enthusiastic about my job

20.c. Time flies when I am working

20.d. I feel physically exhausted at the end of the working day

20.e. I doubt the importance of my work

20.f. In my opinion, I am good at my job

- Always
- Most of the times
- Sometimes
- Rarely
- Never
