# Supplementary material for: General health and working conditions of Flemish primary care professionals
Source: BMC Prim Care. 2023 Jun 29;24:133. doi: 10.1186/s12875-023-02082-w (PMC10308612; doi:10.1186/s12875-023-02082-w)
Supplement: Supplementary file 2 — Additional file 2. [file 12875_2023_2082_MOESM2_ESM.docx]

**SUPPLEMENTARY FILE**

**S2.** Type of profession active in primary care by working conditions (organizational setting, type of contract/employment arrangement and work hours) in %

|  | **% Organizational setting** | | | | | | |
| --- | --- | --- | --- | --- | --- | --- | --- |
| **Type of PC profession** | **solo** | **monodisciplinary team** | | **multidisciplinary team** | **care at home of patients** | **social service** | **other** |
| general practitioner | 21.6 | 33.1 | | 44.6 | 0 | 0 | 0.7 |
| (home) nurse | 5.1 | 19.7 | | 18.0 | 44.9 | 0.6 | 11.8 |
| physiotherapist | 32.2 | 35.6 | | 20.3 | 9.3 | 0 | 2.5 |
| dietician | 22.2 | 11.1 | | 66.7 | 0 | 0 | 0 |
| podiatrist | 25 | 25.0 | | 35 | 0 | 0 | 15 |
| midwife | 15.6 | 46.9 | | 12.5 | 18.8 | 3.1 | 3.1 |
| psychologist | 37.9 | 16.7 | | 30.3 | 3 | 4.5 | 7.6 |
| dentist | 55.6 | 27.8 | | 16.7 | 0 | 0 | 0 |
| social worker | 1.3 | 1.3 | | 9.4 | 15 | 68.8 | 4.4 |
| occupational therapist | 7.5 | 0 | | 7.5 | 32.5 | 32.5 | 20 |
| pharmacist | 45.5 | 50.0 | | 4.5 | 0 | 0 | 0 |
| family care assistent | 0 | 9.1 | | 0 | 81.8 | 0 | 9.1 |
| speech therapist | 33.3 | 25 | | 12.5 | 16.7 | 0 | 12.5 |
| care supporting staff | 0 | 8.3 | | 70.8 | 8.3 | 12.5 | 0 |
| other | 4.9 | 4.9 | | 24.4 | 26.8 | 29.3 | 9.8 |
| N=933 | **% Type of contract/ employment arrangement** | | | | **% Working hours** | | |
|  | **Self-employed** | **Salaried employee** | | **Mix** | **Parttime <35h** | **fulltime 35-40h** | **Fulltime 40+** |
|  |  | **Unlimited duration** | **Limited duration** |  |  |  |  |
| general practitioner | 70.4 | 15.2 | 11.2 | 12.1 | 22.9 | 65.0 | 22.9 |
| (home) nurse | 29.3 | 61.3 | 5.5 | 29.0 | 43.2 | 27.8 | 43.2 |
| physiotherapist | 72.9 | 5.9 | 20.3 | 19.8 | 25.0 | 55.2 | 25.0 |
| dietician | 47.1 | 11.8 | 35.3 | 76.5 | 23.5 | 0.0 | 23.5 |
| podiatrist | 50.0 | 5.0 | 45 | 75.0 | 5.0 | 20.0 | 5.0 |
| midwife | 40.6 | 28.1 | 31.3 | 63.3 | 20.0 | 16.7 | 20.0 |
| psychologist | 47.7 | 4.6 | 43.1 | 78.7 | 14.8 | 6.6 | 14.8 |
| dentist | 100 | 0 | 0 | 55.6 | 27.8 | 16.7 | 27.8 |
| social worker | 0.6 | 95.2 | 0.6 | 39.8 | 56.0 | 4.2 | 56.0 |
| occupational therapist | 5 | 62.5 | 27.5 | 42.5 | 55.0 | 2.5 | 55.0 |
| pharmacist | 34.9 | 55.8 | 4.7 | 16.3 | 34.9 | 48.8 | 34.9 |
| family care assistent | 0 | 90.9 | 9.1 | 60.0 | 40.0 | 0.0 | 40.0 |
| speech therapist | 47.8 | 17.4 | 30.4 | 58.3 | 33.3 | 8.3 | 33.3 |
| care supporting staff | 0 | 96.2 | 3.8 | 64.0 | 28.0 | 8.0 | 28.0 |
| other | 6.3 | 77.1 | 10.4 | 56.3 | 31.3 | 12.5 | 31.3 |
| N= 933 |  |  | |  | N= 934 |  |  |
