# Supplementary material for: General health and working conditions of Flemish primary care professionals
Source: BMC Prim Care. 2023 Jun 29;24:133. doi: 10.1186/s12875-023-02082-w (PMC10308612; doi:10.1186/s12875-023-02082-w)
Supplement: Supplementary file 3 — Additional file 3. [file 12875_2023_2082_MOESM3_ESM.docx]

**SUPPLEMENTARY FILE**

**S3.** Organizational setting and type of contract/employment arrangement by working hours and type of contract/employment arrangement by organizational setting

|  | **Working hours** | |  | **Type of contract/employment arrangement** | | | |
| --- | --- | --- | --- | --- | --- | --- | --- |
|  | parttime <35 | fulltime 35-40 | Fulltime 40+ | self-employed | employed with contract of limited duration | employed with contract of unlimited duration | mix |
| **Organizational setting** | % | % | % | **%** | **%** | % | % |
| solo | 35.22 | 20.75 | 44.03 | 70.25 | 1.27 | 8.86 | 19.62 |
| group-monodisciplinary | 24.47 | 21.28 | 54.26 | 66.49 | 1.05 | 16.23 | 16.23 |
| group-multidisciplinary | 47.85 | 32.54 | 19.62 | 30.88 | 7.35 | 43.14 | 18.63 |
| care at home | 33.33 | 46.54 | 20.13 | 21.74 | 1.24 | 67.08 | 9.94 |
| social service | 43.75 | 50.69 | 5.56 | 0.00 | 2.80 | 95.10 | 2.10 |
| other | 43.64 | 45.45 | 10.91 | 3.51 | 5.26 | 75.44 | 15.79 |
| Total | 37.42 | 34.25 | 28.34 | 36.98 | 3.06 | 45.95 | 14.00 |
| **Type of contract/**  **employment arrangement** | |  |  |  |  |  |  |
| self-employed | 23.26 | 19.03 | 57.70 |  |  |  |  |
| employed with contract of limited duration | 37.93 | 48.28 | 13.79 |  |  |  |  |
| employed with contract of unlimited duration | 40.56 | 51.52 | 7.93 |  |  |  |  |
| mix | 64.00 | 16.80 | 19.20 |  |  |  |  |
| Total | 37.42 | 34.90 | 27.68 |  |  |  |  |

*Chi^2^ tests are performed to see whether the working conditions are significantly related to each other; and all conditions are related at p <0.001. N = 914*
