# Supplementary material for: General health and working conditions of Flemish primary care professionals
Source: BMC Prim Care. 2023 Jun 29;24:133. doi: 10.1186/s12875-023-02082-w (PMC10308612; doi:10.1186/s12875-023-02082-w)
Supplement: Supplementary file 4 — Additional file 4. [file 12875_2023_2082_MOESM4_ESM.docx]

**SUPPLEMENTARY FILE**

**S4.** Correlations between the work engagement scale and the employment quality scales

|  | work life balance | | proper rewards | carreer opportunities | job recognition | supportive relations with colleagues | job security | perceived employability |
| --- | --- | --- | --- | --- | --- | --- | --- | --- |
| work engagement | 0.174*** | | 0.020 | 0.170*** | 0.303*** | 0.166*** | 0.084* | -0.005 |
| work life balance | 1 | | 0.307*** | 0.173*** | 0.267*** | 0.131*** | 0.020 | -0.019 |
| proper rewards |  | | 1 | 0.355*** | 0.367*** | 0.075* | -0.028 | -0.027 |
| carreer opportunities |  | |  | 1 | 0.353*** | 0.113*** | 0.036 | 0.066* |
| job recognition |  | |  |  | 1 | 0.140*** | 0.131*** | 0.031 |
| supportive relations  with colleagues |  |  | |  |  | 1 | 0.152*** | 0.174*** |
| job security |  | |  |  |  |  | 1 | 0.187*** |

*Pearson’s correlations tests; * p < 0.01 **p < 0.05 *** p < 0.001; N = 826*
